# Supplementary material for: Estimation of time-varying causal effects with multivariable Mendelian randomization: some cautionary notes
Source: Int J Epidemiol. Author manuscript; Available in PMC 2023 Jun 8. (PMC10244034; doi:10.1093/ije/dyac240)
Supplement: Supplementary File [file EMS163168-supplement-Supplementary_File.pdf]

Supplementary material: Estimation of time-varying causal effects with multivariable Mendelian randomization: some cautionary notes

Haodong Tian [1,*]
Stephen Burgess [1,2]

[1] MRC Biostatistics Unit, University of Cambridge, Cambridge, UK

[2] Cardiovascular Epidemiology Unit,
Department of Public Health and Primary Care,
University of Cambridge, Cambridge, UK

[*] Corresponding author. MRC Biostatistics Unit, University of Cambridge,
East Forvie Building, Forvie Site, Robinson Way, Cambridge, CB2 0SR, UK.
Email: haodong.tian@mrc-bsu.cam.ac.uk

Contents

Supplementary Text **2**
Text S1: General illustration of MVMR estimates 2
Text S2: Asymptotic estimates with linear time-varying instrument effects . . 3
Text S3: Extended situation for the cumulative effect 3
Text S4: Supplementary simulation 4

Supplementary Tables **6**
Table S1: Effect parameters used for simulation 6
Table S2: Instrument strength information in simulation 7
Table S3: Instrument strength information of the real example 8

Supplementary Figures **9**
Figure S1: Individual trajectory 9
Figure S2: Demonstration diagram for the UK Biobank example 10
Figure S3: Simulation results with stronger instruments 11
Figure S4: Scatter plots of the instrument-exposure associations 12
Figure S5: Individual trajectory of Scenario 4 13
Figure S6: Simulation results of Scenario 4 14

Supplementary Text

Text S1: General illustration of MVMR estimates

This appendix gives the property and the interpretation of MVMR estimates with general instrument effects under the continuous-time model. Assume the structural equation is

$$Y = \int_0^T \beta(t)X(t)dt + \epsilon \tag{S1}$$

and the time-varying exposure can be expressed as

$$X(t) = \sum_{j=1}^{J} \alpha_j(t)G_j + v(t) \qquad t \in [0, T] \tag{S2}$$

where J is the number of valid instruments and $cov(G_j, v(t)) = 0$ for any j and t. For convenience we call $\alpha_j(t)$ the instrument effect on the exposure at time t of the j-th instrument, though they do not necessarily have the causal interpretation. Assume the K $(1 \leq K \leq J)$ distinct increasing measured timepoints for the exposure is $\{t_1, \ldots, t_K\}$, which corresponds to the K exposures in MVMR; that is, $\{X_k = X(t_k); k = 1, \ldots, K\}$. The MVMR estimates are equivalently and consistently derived by using either IVW (also for summary data), 2SLS or g-estimation. For IVW, it is to fit the weighted regression

$$\hat{\boldsymbol{\theta}} = \boldsymbol{A}\boldsymbol{\beta} + \boldsymbol{\epsilon} \tag{S3}$$

where $\{\hat{\boldsymbol{\theta}}\}_j = \hat{\theta}_j$, the estimated genetic association with the outcome of the j-th instrument; $\{\boldsymbol{A}\}_{j,k} = \hat{\alpha}_j(t_k)$, the estimated instrument effect at the measured timepoint t_k of the j-th instrument; $\boldsymbol{\epsilon} \sim \mathcal{N}(\boldsymbol{0}, \boldsymbol{\Sigma})$ where $\boldsymbol{\Sigma}$ is the estimated variance matrix for $\hat{\boldsymbol{\theta}}$; Here we assume there is no measurement error of the instrument effects and the variance matrix $\boldsymbol{\Sigma}$. $\hat{\boldsymbol{\beta}}$ are the MVMR estimates one wishes to interpret, and it satisfies

$$\begin{aligned}
\hat{\boldsymbol{\beta}} =& (\boldsymbol{A}^T\boldsymbol{\Sigma}^{-1}\boldsymbol{A})^{-1}\boldsymbol{A}^T\boldsymbol{\Sigma}^{-1}\hat{\boldsymbol{\theta}} \\
=& (\boldsymbol{A}^T\boldsymbol{\Sigma}^{-1}\boldsymbol{A})^{-1}\boldsymbol{A}^T\boldsymbol{\Sigma}^{-1}\left(\int_0^T \beta(t)\boldsymbol{\alpha}(t)dt + \boldsymbol{e}\right) \\
=& \int_0^T \beta(t)(\boldsymbol{A}^T\boldsymbol{\Sigma}^{-1}\boldsymbol{A})^{-1}\boldsymbol{A}^T\boldsymbol{\Sigma}^{-1}\boldsymbol{\alpha}(t)dt + (\boldsymbol{A}^T\boldsymbol{\Sigma}^{-1}\boldsymbol{A})^{-1}\boldsymbol{A}^T\boldsymbol{\Sigma}^{-1}\boldsymbol{e} \\
=& \int_0^T \beta(t)\boldsymbol{w}(t)dt + (\boldsymbol{A}^T\boldsymbol{\Sigma}^{-1}\boldsymbol{A})^{-1}\boldsymbol{A}^T\boldsymbol{\Sigma}^{-1}\boldsymbol{e}
\end{aligned} \tag{S4}$$

where $\{\boldsymbol{\alpha}(t)\}_j = \alpha_j(t)$ and the second equation is approximated by the asymptotic normality with respect to the true structural equation (S1) and the exposure equation (S2), and $\boldsymbol{e} \xrightarrow{D} \mathcal{N}(\boldsymbol{0}, \boldsymbol{\Sigma})$. It means the MVMR estimates represent weighted cumulative effects with the weighting function $\boldsymbol{w}(t) = (\boldsymbol{A}^T\boldsymbol{\Sigma}^{-1}\boldsymbol{A})^{-1}\boldsymbol{A}^T\boldsymbol{\Sigma}^{-1}\boldsymbol{\alpha}(t)$. Note that the weight $\boldsymbol{w}(t)$ is obtained equivalently by fitting the auxiliary regression

$$\boldsymbol{\alpha}(t) = \boldsymbol{A}\boldsymbol{w}(t) + \boldsymbol{\epsilon} \qquad \boldsymbol{\epsilon} \sim \mathcal{N}(\boldsymbol{0}, \boldsymbol{\Sigma}) \tag{S5}$$

The implication of $\boldsymbol{w}(t)$ is therefore the internal association of the instrument effects at the measured timepoints $\{t_k; k = 1, \ldots, K\}$ and the instrument effects at the timepoint

t among the instruments. Note that the mean of a single (e.g. the k-th) estimate of the MVMR can be non-zero even at the timepoint over which the exposure has zero effect (i.e. $\beta(t_k) = 0$) because the instrument effects at t_k may be correlated with the instrument effects at other timepoints over which $\beta(t) \neq 0$. In addition, a single MVMR estimate $\boldsymbol{\beta}_j$ should be regarded as the weighted cumulative effect with the form $\int_0^T \beta(t)\boldsymbol{w}_j(t)dt$ where the weight function is determined by the internal association of the instrument effects (i.e $\alpha(\cdot)$) over timepoints among the instruments, which makes the interpretation of MVMR difficult and even impossible as one may not have enough information about the time-varying instrument effect.

In practice, one draws a test to a single (e.g. the k-th) estimate of MVMR to make a relevant causal conclusion. For $H_0 : \beta_k = 0$, it is to check the rejection rule

$$\mathcal{R} = \left\{ \left| \frac{\hat{\beta}_k}{\sqrt{(\boldsymbol{A}^T\boldsymbol{\Sigma}^{-1}\boldsymbol{A})^{-1}_{k,k}}} \right| > z_s \right\} \tag{S6}$$

where z_α represents the critical value with the significance level s. Even if there is no measurement error of $\boldsymbol{A}$ and $\boldsymbol{\Sigma}$ and we have sufficient samples, according to the equation (S4) and the asymptotic normality $\hat{\beta}_k \sim \mathcal{N}(\int_0^T \beta(t)\boldsymbol{w}_k(t)dt, (\boldsymbol{A}^T\boldsymbol{\Sigma}^{-1}\boldsymbol{A})^{-1}_{k,k})$, we have

$$P(\mathcal{R}) = P\left(\left| \frac{\hat{\beta}_k}{\sqrt{(\boldsymbol{A}^T\boldsymbol{\Sigma}^{-1}\boldsymbol{A})^{-1}_{k,k}}} \right| > z_s \right) = P(|Z_{\mathbb{E}(\hat{\beta}_k)/\sqrt{(\boldsymbol{A}^T\boldsymbol{\Sigma}^{-1}\boldsymbol{A})^{-1}_{k,k}}}| > z_s) > s \tag{S7}$$

when $\mathbb{E}(\hat{\beta}_1) = \int_0^T \beta(t)\boldsymbol{w}_k(t)dt \neq 0$ and where Z_x represents the normal distribution with mean x and variance 1. It tells that the test regarding a single MVMR estimate may have the inflated type I error and therefore invalid even the null hypothesis that $\beta(t) = 0$ over a particular time region holds.

Text S2: Asymptotic estimates with linear time-varying instrument effects

Suppose the instrument effects on the exposure of all the instruments vary linearly over time, that is

$$\alpha_j(t) = a_j + b_j t \qquad j = 1, \dots, J \tag{S8}$$

Let the number of measured timepoints be two (otherwise, $\boldsymbol{A}$ is singular) and they are t_1 and t_2. It is easy to know via interpolation or extrapolation for each instrument that

$$\boldsymbol{\alpha}(t) = w_1(t)\boldsymbol{\alpha}(t_1) + w_2(t)\boldsymbol{\alpha}(t_2) \tag{S9}$$

where $w_1(t) = (t_2 - t)/(t_2 - t_1)$ and $w_2(t) = (t - t_1)/(t_2 - t_1)$. In the exact-identification case where $\boldsymbol{A} = (\boldsymbol{\alpha}(t_1)\,\boldsymbol{\alpha}(t_2)) \in \mathbb{R}^{2\times 2}$ is of full rank, we have

$$\hat{\boldsymbol{\beta}} = \hat{\boldsymbol{A}}^{-1}\hat{\boldsymbol{\theta}} \xrightarrow{p} \boldsymbol{A}^{-1}\int_0^T \beta(t)\boldsymbol{\alpha}(t)dt = \int_0^T \beta(t)\boldsymbol{A}^{-1}\boldsymbol{\alpha}(t)dt = \int_0^T \beta(t)\boldsymbol{w}(t)dt \tag{S10}$$

where $\boldsymbol{w}(t) = (w_1(t)\,w_2(t))^T$. It means

$$\operatorname*{plim}_{n\to\infty} \hat{\beta}_1 = \int_0^T \beta(t)\frac{t_2 - t}{t_2 - t_1}dt \qquad \operatorname*{plim}_{n\to\infty} \hat{\beta}_2 = \int_0^T \beta(t)\frac{t - t_1}{t_2 - t_1}dt \tag{S11}$$

Text S3: Extended situation for the cumulative effect

We consider the extended situation where the time-varying instrument effects can be expressed as the polynomial with a degree of p ($p \geq 0$). That is,

$$\alpha_j(t) = \sum_{k=0}^{p} a_{k,j} t^k \tag{S12}$$

where $a_{k,j}$ represents the $(k+1)$-th polynomial coefficients for the j-th instrument. Assume we have $p+1$ distinct and increasing measured timepoints $t_1, \ldots, t_{p+1}$. We consider the exact-identification case that the number of valid instruments is $p+1$ and $\boldsymbol{A}$ is of full rank. For each instrument, the polynomial coefficients can be represented by the instrument effects at the measured timepoints; that is

$$\boldsymbol{a}_j = \boldsymbol{V}^{-1}\alpha_j(\boldsymbol{t}_m) \qquad j = 1, \ldots, p+1 \tag{S13}$$

where $\boldsymbol{a}_j = (a_{0,j}\, a_{1,j} \cdots a_{p,j})^T$, $\boldsymbol{t}_m = (t_1\, t_2 \cdots t_{p+1})^T$ are the vector of the measured timepoints and $\alpha_j(\boldsymbol{t}_m) \in \mathbb{R}^{p+1}$, and $\{\boldsymbol{V}\}_{l,m} = t_l^{m-1}$ where $1 \leq l, m \leq p+1$. $\boldsymbol{V}$ is a squared Vandermonde matrix. Note that $\boldsymbol{V}^{-1}$ exists due to the squared Vandermonde matrix property that its determinant is non-zero if the measured timepoints are distinct. Hence we can express the instrument effect at any timepoint by the instrument effects at the measured timepoints for each instrument as

$$\alpha_j(t) = \boldsymbol{p}_t^T \boldsymbol{a}_j = \boldsymbol{p}_t^T \boldsymbol{V}^{-1}\alpha_j(\boldsymbol{t}_m) \qquad j = 1, \ldots, p+1 \tag{S14}$$

where $\boldsymbol{p}_t = (1\ t\ \cdots\ t^p)^T$. Since $\boldsymbol{p}_t^T \boldsymbol{V}^{-1}$ is independent of j, and it is easy to know

$$\boldsymbol{p}_t^T \boldsymbol{V}^{-1} \boldsymbol{A}^T = \boldsymbol{\alpha}^T(t) \tag{S15}$$

Set $\boldsymbol{e} = (1 \ldots 1)^T \in \mathbb{R}^{p+1}$. The total sum of MVMR estimates, $\hat{\boldsymbol{\beta}}^T \boldsymbol{e}$, will have

$$\hat{\boldsymbol{\beta}}^T \boldsymbol{e} = \hat{\boldsymbol{\theta}}^T \hat{\boldsymbol{A}}^{-T} \boldsymbol{e} \xrightarrow{p} \int_0^T \beta(t)\boldsymbol{\alpha}^T(t)dt\boldsymbol{A}^{-T}\boldsymbol{e} = \int_0^T \beta(t)\boldsymbol{\alpha}^T(t)\boldsymbol{A}^{-T}\boldsymbol{e}dt \tag{S16}$$

Note that

$$\boldsymbol{\alpha}^T(t)\boldsymbol{A}^{-T}\boldsymbol{e} = \boldsymbol{p}_t^T \boldsymbol{V}^{-1}\boldsymbol{e} = \boldsymbol{p}_t^T \boldsymbol{o} \tag{S17}$$

where the first equation is given by equation (S15), and $\boldsymbol{o} = \boldsymbol{V}^{-1}\boldsymbol{e} \in \mathbb{R}^{p+1}$. Since the first column element of $\boldsymbol{V}$ is always one, it is easy to know $\boldsymbol{o} = (1\,0 \ldots 0)^T$ and $\boldsymbol{\alpha}^T(t)\boldsymbol{A}^{-T}\boldsymbol{e} = 1$. Therefore,

$$\operatorname*{plim}_{n\to\infty} \sum_{k=1}^{p+1} \hat{\beta}_k = \operatorname*{plim}_{n\to\infty} \hat{\boldsymbol{\beta}}^T \boldsymbol{e} = \int_0^T \beta(t)dt \tag{S18}$$

The direct application of this conclusion is the two measured timepoints example mentioned above. Besides, when there is only one measured timepoint (i.e. the univariate Mendelian randomization), the MR estimate can be interpreted as the cumulative effect if the instrument effects are polynomial of the degree $p = 0$, which is equivalent to require that all the instrument effect is constant (i.e., time-invariant).

Text S4: Supplementary simulation

We add an additional simulation scenario, denoted by Scenario 4, in which the genetic effects on the exposure follow a model in which genetic variants affect the exposure at one timepoint but not at another (that is, they switch on and off at a specific timepoint). The time-varying model is similar to the Scenario 3A-3D

$$X(t) = \sum_{j=1}^{30} \alpha_j(t)\, G_j + U_0 + U(t) + \epsilon_X(t) \qquad t \in [0, 50] \tag{S19}$$

$$Y = \int_0^{50} \beta(t) X(t) dt + U_0 + U(50) + \epsilon_Y(50) \tag{S20}$$

where for $j = 1, 3, 5, \ldots, 29$,

$$\alpha_j(t) = 0.1 \text{ if } t \leq \frac{50}{16}\frac{j+1}{2}\text{, and 0 otherwise} \tag{S21}$$

and for $j = 2, 4, 6, \ldots, 30$,

$$\alpha_j(t) = 0.1 \text{ if } t > \frac{50}{16}\frac{j}{2}\text{, and 0 otherwise} \tag{S22}$$

We also consider the effect $\beta(t)$ of Scenario 2A (null effect until time 40 and positive effect thereafter) and run the MVMR analysis with four sets of timepoints; They are $(10, 50)$, $(20, 50)$, $(40, 50)$ and $(20, 40)$, denoted by Scenario 4A, 4B, 4C and 4D. The trajectory for the exposure of one individual, along with the instrument and confounding effects, is shown in Supplementary Figure S5. The fitting results by MVMR are given in Supplementary Figure S6.

Supplementary Tables

Scenario	A_1	A_2	A_3	A_4
1	$\mathcal{N}(0, 0.05^2)$	$\mathcal{N}(0, 0.15^2)$	$\mathcal{N}(3, 0.01^2)$	$\mathcal{N}(0, 1^2)$
1 (with stronger instruments)	$\mathcal{N}(0, 0.1^2)$	$\mathcal{N}(0, 0.3^2)$	$\mathcal{N}(3, 0.01^2)$	$\mathcal{N}(0, 1^2)$
$2A$	$\mathcal{N}(0, 0.05^2)$	$\mathcal{N}(0, 0.05^2)$	$\mathcal{N}(0, 1^2)$	$\mathcal{N}(0, 1^2)$
$2B$	$\mathcal{N}(0, 0.05^2)$	$\mathcal{N}(0, 0.15^2)$	$\mathcal{N}(3, 0.01^2)$	$\mathcal{N}(0, 1^2)$
$2C$	$\mathcal{N}(0, 0.05^2)$	$\mathcal{N}(0, 0.15^2)$	$\mathcal{N}(0.1, 0.01^2)$	$\mathcal{N}(0, 1^2)$

Supplementary Table S1: The independent normal distribution used in the effect parameters (A_1, A_2, A_3, A_4 in Equation (2)) of genetic variants for each simulation study scenario. $\mathcal{N}(\mu, \sigma^2)$ representing the normal distribution with the mean μ and the variance σ^2.

Scenario	timepoint	R^2	F	conditional F
$1A$	10	0.099	36.4	20.6
	50	0.106	39.2	22.2
$1B$	10	0.099	36.4	14.0
	40	0.129	49.4	31.8
	50	0.106	39.2	14.6
$1C$	15	0.120	45.2	34.6
	30	0.147	57.3	43.9
$1D$	15	0.120	45.2	46.7
	50	0.106	39.2	40.5
$2A$	10	0.038	13.2	11.8
	50	0.027	9.1	8.1
$2B$	10	0.116	43.5	28.9
	50	0.157	62.0	41.2
$2C$	10	0.097	35.8	20.9
	50	0.086	31.5	18.4
$3A$	10	0.036	12.3	5.3
	50	0.055	19.4	8.3
$3B$	20	0.037	12.7	2.5
	50	0.055	19.4	3.8
$3C$	40	0.048	16.7	0.3
	50	0.055	19.4	0.3
$3D$	20	0.037	12.7	1.6
	40	0.048	16.7	2.1

Supplementary Table S2: Average values of instrument strength in the various simulation study scenarios. R^2 represents the average proportion of variance in the exposure explained by the genetic variants. F and conditional F is the corresponding univariate and conditional F statistic value.

First time period	R^2	F	Conditional F (for first time period)	Conditional F (for second time period)
41-46	0.027	12.2	1.04	1.10
42-47	0.025	11.8	0.90	0.91
43-48	0.024	11.5	0.90	0.92
44-49	0.023	11.7	0.99	1.04
45-50	0.021	11.1	1.04	1.11
46-51	0.021	11.3	1.13	1.26
47-52	0.023	12.5	1.25	1.46
48-53	0.023	13.4	1.19	1.33
49-54	0.023	13.8	1.21	1.37
50-55	0.024	14.3	1.13	1.26
51-56	0.024	15.1	1.05	1.09
52-57	0.022	14.4	1.14	1.17
53-58	0.021	13.8	1.23	1.27
54-59	0.021	14.6	1.22	1.24
55-60	0.021	15.1	1.11	1.11

Supplementary Table S3: Instrument strength values for multivariable Mendelian randomization analysis of the real example with different time periods. The first conditional F statistic is for the BMI over the first time period as indicated. The second conditional F statistic is for the BMI over the fixed time period from age 60 to 65. R^2 represents the proportion of variance in the exposure explained by the genetic variants. F is the corresponding conventional F statistic value. The R^2 and F value for the second time period is 0.019 and 18.5.

Supplementary Figures

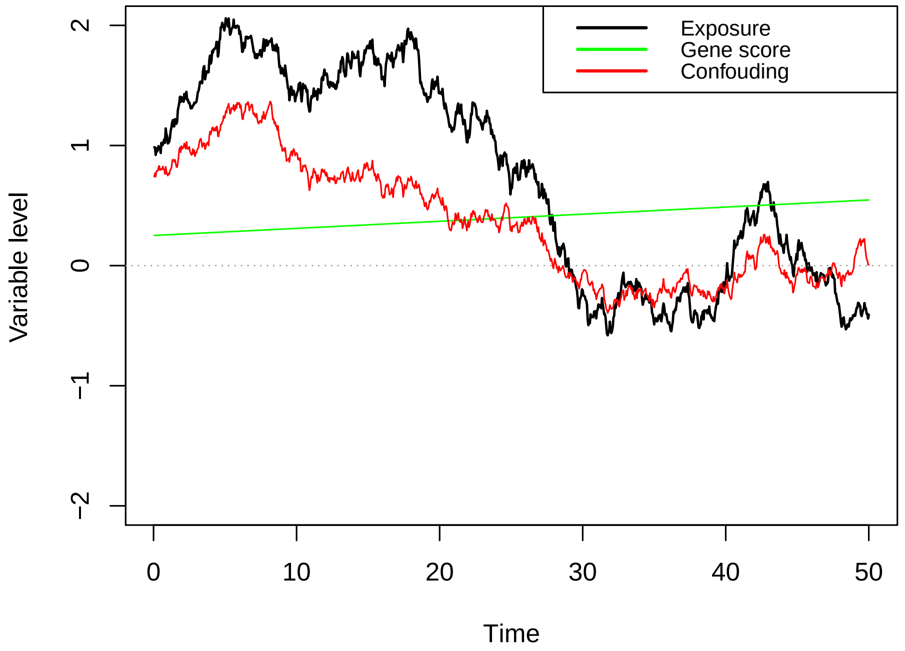

Supplementary Figure S1: One individual trajectory based on the model (7) consisting of the exposure values (black curve), the genetic effect on the exposure (green curve) and the confounding effect (red curve) over time.

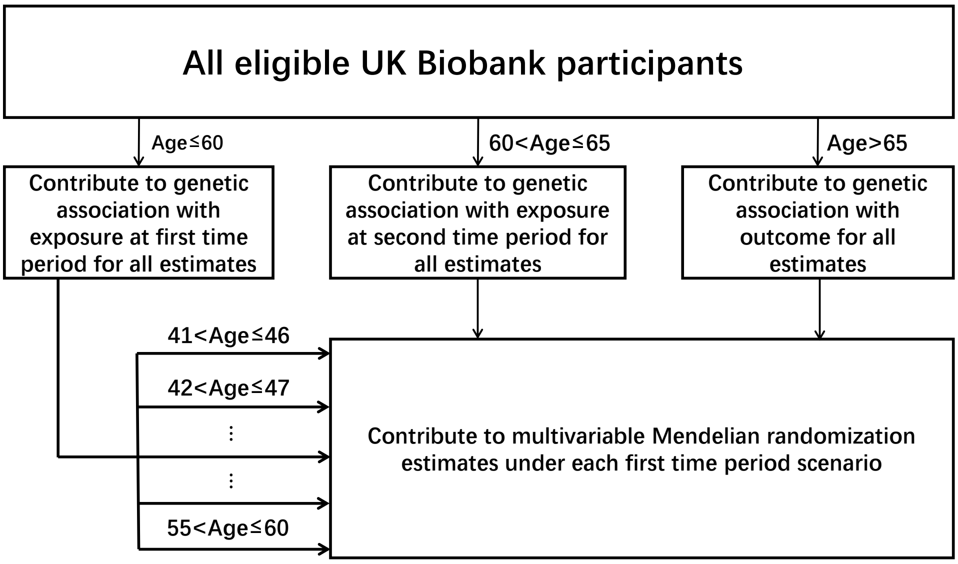

Supplementary Figure S2: The diagram demonstrating the multivariable Mendelian randomization fitting with the UK Biobank data.

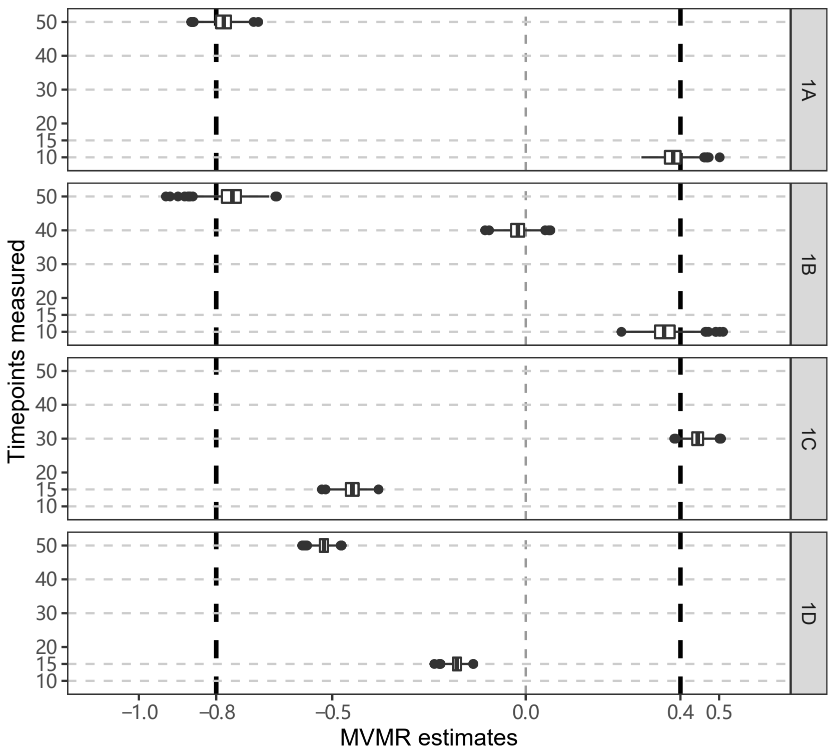

Supplementary Figure S3: Simulation results with stronger instruments (the average proportion of variance in the exposure explained was around 30%) when the outcome is affected discretely by the exposure at specific time-points. Boxplots of multivariable Mendelian randomization (MVMR) estimates with risk factors taken as the exposure measure at different measured timepoints – Scenario 1A: times 10 and 50; Scenario 1B: 10, 40, 50; Scenario 1C: 15, 30; Scenario 1D: 15, 50. Box indicates lower quartile, median, and upper quartile; error bars represent the minimal and maximal data point falling in the 1.5 interquartile range distance from the lower/upper quartile; estimates outside this range are plotted separately. The true effects are $\beta_1 = 0.4$ at time 10 and $\beta_2 = -0.8$ at time 50 (black dashed lines).

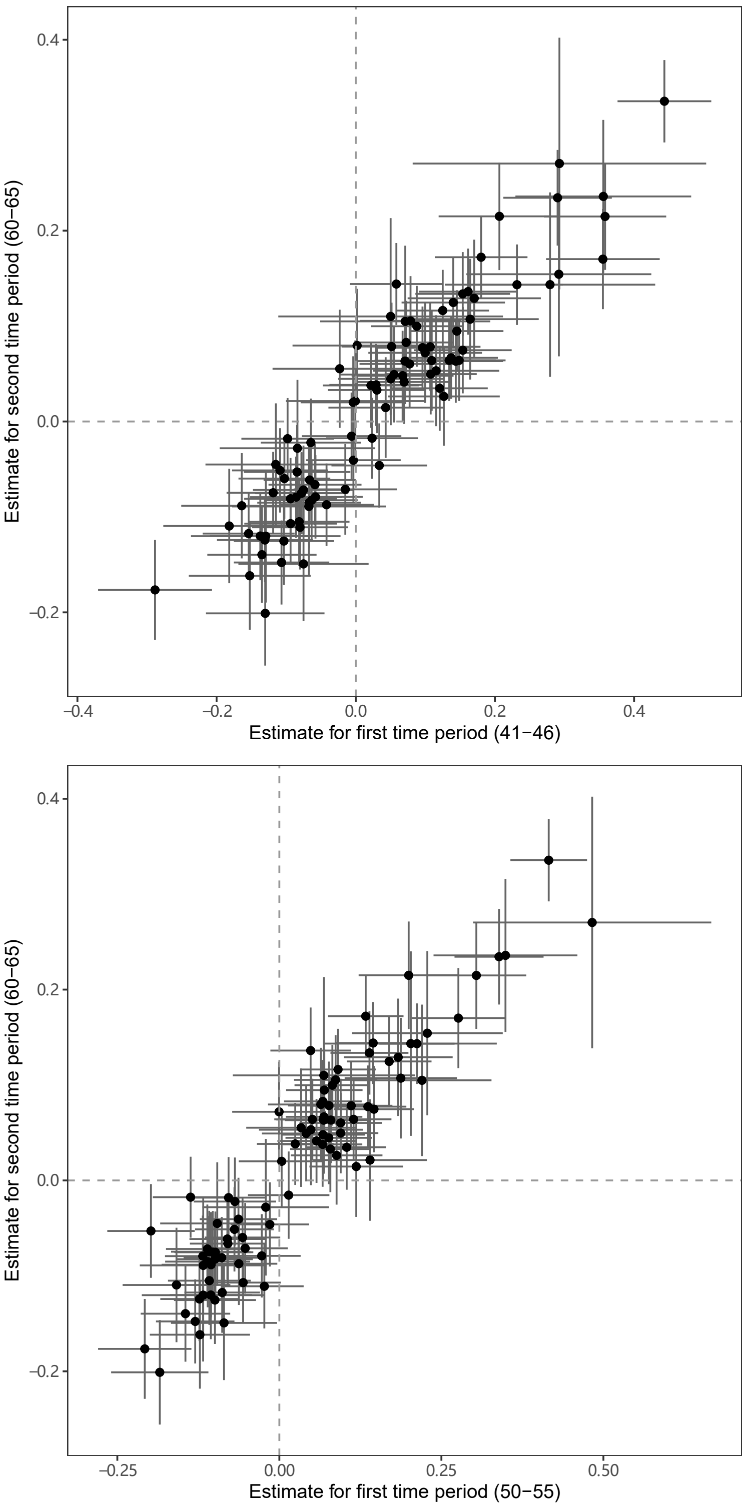

Supplementary Figure S4: Scatter plots of the instrument-exposure associations at two different time periods for 93 genetic variants. In the top case, the two time periods in which the exposure was measured are 41-46 and 60-65. In the bottom case, the two time periods are 50-55 and 60-65. The error bar represents the 95% confidence interval for the corresponding instrument-exposure association estimate.

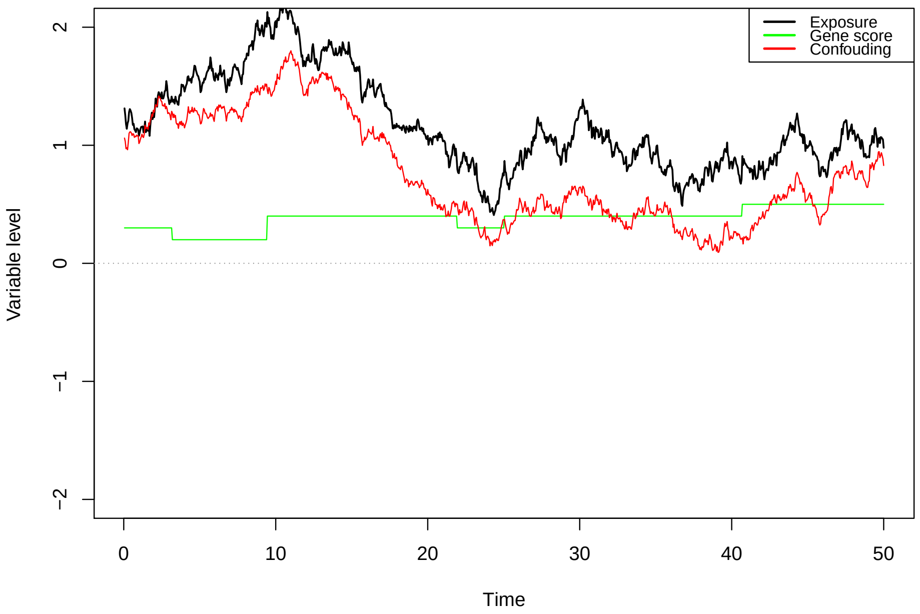

Supplementary Figure S5: One individual trajectory based on the model (S21)-(S22) consisting of the exposure values (black curve), the genetic effect on the exposure (green curve) and the confounding effect (red curve) over time.

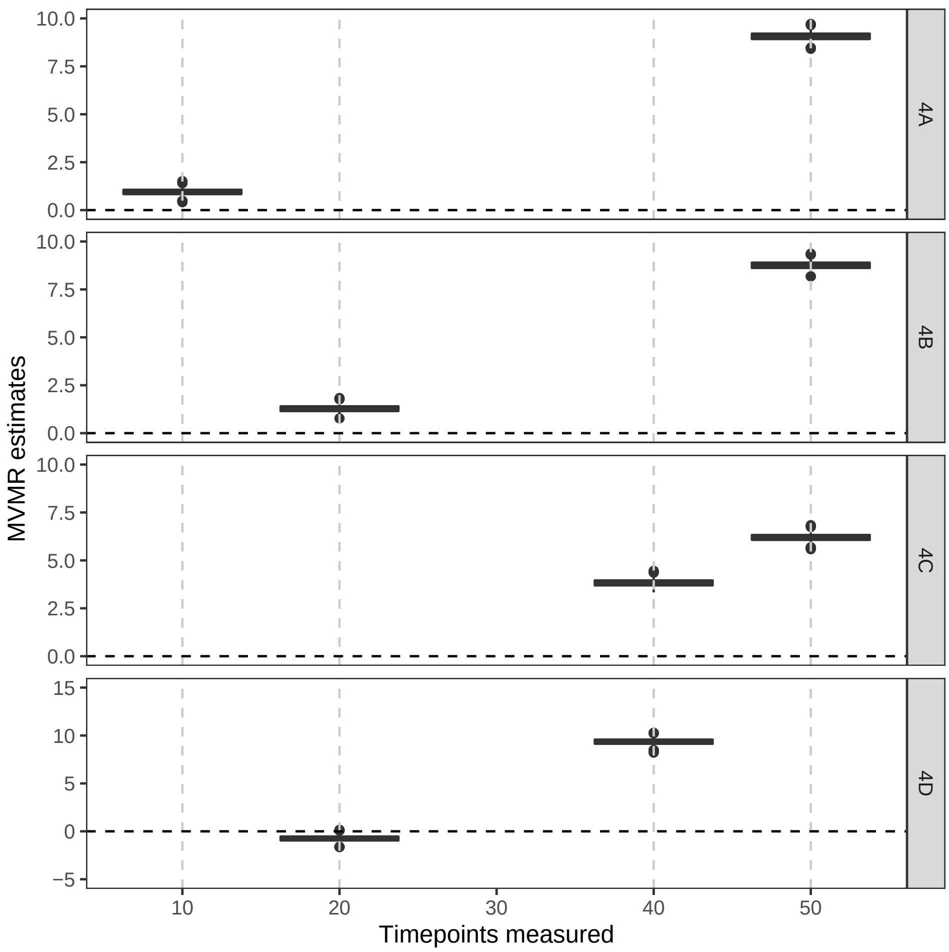

Supplementary Figure S6: Simulation results when the outcome is affected continuously by the exposure across time and the genetic effects on the exposure switch on and off at specific time. Boxplots of multivariable Mendelian randomization (MVMR) estimates with risk factors taken as the exposure measure at different measured timepoints: $(10, 50)$, $(20, 50)$, $(40, 50)$ and $(20, 40)$, denoted by Scenario 4A, 4B, 4C and 4D. The underlying continuous effect is as in Scenario 2A: null effect until time 40, positive effect thereafter. Box indicates lower quartile, median, and upper quartile; error bars represent the minimal and maximal data point falling in the 1.5 interquartile range distance from the lower/upper quartile; estimates outside this range are plotted separately.
